# Is There a Link Between Frequency of Dreams, Lucid Dreams, and Subjective Sleep Quality?

**DOI:** 10.3389/fpsyg.2020.01290

**Published:** 2020-06-25

**Authors:** Nicolas Ribeiro, Yannick Gounden, Véronique Quaglino

**Affiliations:** CRP-CPO, EA 7273, Université de Picardie Jules Verne, Amiens, France

**Keywords:** dreaming, lucid, consciousness, frequency, prevalence

## Abstract

A lucid dream is a dream in which one is conscious of dreaming and can possibly control the dream or passively observe its unfolding. Frequencies of lucid dreaming (LD), dream with awareness, and dream with actual control were previously investigated in a French student population. As a student population usually differs on oneiric and sleep characteristics (such as sleep quality) from the general population, more investigations were needed. Additionally, it is yet unresolved if LD is related to one’s overall sleep quality. This study aims at describing and comparing dream experience frequencies (dream, lucid dreams, awareness, and control) and sleep quality assessed with the Pittsburgh Sleep Quality Index (PSQI) among students (*n* = 274) and in a general population sample (*n* = 681). It also aims at evaluating if dream experience frequencies can predict sleep quality across these two samples. Predictive models of PSQI score controlling for age and gender were not significant in the student group while they were all marginally predictive for the general population. However, none of these models showed that the frequency of dream experiences could actually help predict the quality of sleep as the significance of the model was carried over only by the gender variable. These results are discussed in line with previous studies on LD frequencies. Several methodological adjustments for future study are proposed.

## Introduction

Lucid dreaming (LD) is defined as a dream in which the dreamer, while dreaming, is aware that he or she is dreaming. In such a dream, the dreamer has the possibility to control the dream content or to observe the dream unfold passively (Schredl and Erlacher, 2004). A definition of LD has gained popularity in the scientific literature over the last two decades that stipulates that “*In lucid dreams, one has awareness that one is dreaming during the dream. Thus it is possible to wake up deliberately, or to influence the action of the dream actively, or to observe the course of the dream passively*” (Schredl and Erlacher, 2004).

Lucid dreaming can be apprehended in different ways. For instance, LD can be conceived as a hybrid state of consciousness in which subjective experience is seen as similar to wake like functioning while the dreamer remains asleep. The extents of this theory are that insight (awareness) concerning the dream state and volitional control are features of wake functioning and therefore the sign of an atypical functioning when occurring in dreams (Voss et al., 2009). Within the context of this conception, LD is considered as an abnormality which is a consequence of a shift in brain activity that alters normal REM sleep toward waking functioning (that feature insight and ego) while the dreamer still exhibits atonia and rapid eye movement burst (Voss et al., 2009, 2015). The hybrid theory has recently been put forward to posit that an increased frequency of LD could potentially dysregulate sleep and have an incidence on one’s sleep quality (Vallat and Ruby, 2019). This conception has evolved progressively in favor of other views that invite to consider more contrasts or gradations between states of consciousness (see for instance the Space of Consciousness Model from Voss et al., 2015). The continuum perspective is another way to consider LD in which awareness or control are not specifically attributed to wake or dream-like functioning (Stumbrys, 2011). In such conception, the heightened REM brain activity that is shown when one is LD presents no strong rationales to unfavorably influence typical sleep quality.

The main question that will be addressed in this study is the existence of an influential link between the frequency of dream experiences and the overall sleep quality. Determining the existence of a detrimental or beneficial effect of LD on sleep quality can provide information about what it is and how it should be addressed when evaluated in our research. Previous studies have obtained results concerning this relation between LD and sleep parameters. For example, Denis and Poerio (2017) investigated LD in an online survey based on a large population sample (18–82 years, *n* = 1,928). Their results have highlighted correlations between LD and sleep paralysis episodes. No more correlation between LD and the other sleep quality parameters evaluated with the eight-item Sleep Condition Indicator (SCI) were found (Espie et al., 2014; Denis and Poerio, 2017). Alternatively, a psychology student-based sample (*n* = 187, 73% women) proposed two questionnaires and a sleep diary across a period of 2 months (Aviram and Soffer-Dudek, 2018). LD frequency obtained using a 5-item scale in the first questionnaire was weakly (*r* = 15) associated to a poorer sleep quality as reported by the global sleep assessment questionnaire (Aviram and Soffer-Dudek, 2018). Specifically, only the frequency of deliberate attempts to experience the lucid dream state (through techniques designed to increase the likelihood of LD) was associated with a sleep problem among the five items (momentary frequency, prolonged frequency, spontaneous frequency, frequency of attempt, and frequency of success). In another study, the relationship between LD frequency and sleep quality was investigated in two samples: students (*n* = 442) and general population (*n* = 1,380) (Schadow et al., 2018). In this study of Schadow et al. (2018), sleep quality was assessed over the course of 2 weeks for the student sample. A composite score on the perceived quality of sleep was calculated on 11 items based on the SF-B sleep questionnaire (Görtelmeyer, 1986, cited in Schadow et al., 2018). For the general population group, perceived sleep quality was assessed using a general questionnaire based on the last month. LD frequency was calculated using the same LD scale as the one in the present study in the two groups. LD was related to a poorer sleep quality in both groups, but this relation disappeared when controlling for nightmare frequency. Finally, a recent diary study performed for 5 weeks that included 149 participants showed that having a lucid dream during a night can be correlated with a higher feeling of being refreshed at wake, contrasting the view of LD as detrimental (Schredl et al., 2020).

Considering these contrasted results, pursuing these investigations of how LD influence sleep quality is a necessity. For this aim, general sleep quality characteristics can be assessed by subjective reports using the Pittsburgh Sleep Quality Index (PSQI; Buysse et al., 1989). The PSQI could be valuable as it investigates sleep quality over the last month and propose a score calculated over seven components (sleep latency, sleep duration, sleep efficiency, sleep disturbances, medications, and daytime dysfunction). Concerning dream experiences frequencies (typical dream, lucid dream, dream with awareness, and dream with control), a previous study revealed that they are susceptible to relate differently with sleep characteristics, precisely parasomnias correlated with dream control frequency only when these correlations were not found for LD evaluated with a definition or with the question of dream awareness (Ribeiro et al., 2016). Thus, relying on the same methodology for assessing dream experience frequencies as the aforementioned study could reveal a specific relationship with sleep quality that would not have been apparent otherwise. Comparing these two sample types should be done while controlling for age and gender as these factors are supposed to influence dream frequency and sleep quality (Schredl and Reinhard, 2008).

There are few up-to-date investigations of sleep quality and dream experience frequencies among French students; a previous study was performed on 1,137 students (Vallat et al., 2018). The students who were selected in the study of Vallat et al. (2018) were those who did not report any sleep disorders; as a consequence of this selection, the sleep quality possibly have been overestimated and it could be a need to extend such type of study to a more open to everybody sample without any precise inclusion criteria. To our knowledge, there are no studies describing and comparing the results of dream experiences frequency and sleep quality obtained with French students and with a general population sample using the same methodology. However, college students commonly exhibit sleep difficulty singularities in terms of subjective sleep quality (Lund et al., 2010; Lopes et al., 2013). Defining sleep quality on French students is of high importance as, for instance, it could be informative in terms of prevention strategy.

In another scope, continuing to define what LD is and how it is represented in different populations remains critical given its significance for the understanding of consciousness (Noreika et al., 2010). To our knowledge, it is not yet known what causes the difference in the frequency of dream experiences observed in several studies (see Table 1 on Ribeiro et al., 2016); therefore, using an unselected general sample to complete observation previously made on students is of importance.

The aim of the present study is to evaluate if dream experience frequencies (dream, lucid dreams, awareness, and control dreams) are related to subjective sleep quality (assessed with a total score of the PSQI). Within this scope we will describe dream experience frequencies and subjective sleep quality. In light of previous study, we hypothesize that sleep quality will be influenced marginally by atypical dream experiences frequencies.

## Materials and Methods

### Participants

Two samples of French participants were included in this study (final sample *n* = 955). The student sample was recruited using the university’s online communications and social networks. They were 274 (219 women) undergraduate students with a mean age of 21.33 ± 3.27 years ranging from 19 to 52 years. The population-based sample was recruited using online communication and the university students relayed the call for participation. No selection criterium was indicated. They were 681 with a mean age of 34.63 ± 15.56 years ranging from 19 to 89 years. There were 400 women and 241 men. Both groups completed the questionnaire from January to February 2020. Out of 1,054 participants, 99 were excluded from analysis as participants indicated “No” or “Rather not” to the following question: “*Does this questionnaire contain answers that reflect (your) actual reality?*”

In a study investigating subjective sleep parameters, the gender factor can rationally be supposed to influence the results (Schredl and Piel, 2003; see Schredl and Reinhard, 2008). Dream experiences frequencies and score of the PSQI have been compared across genders. In the student group, comparison was significant for dream recall frequencies (*p* = 0.018). In the general population, the comparison was significant for dream recall frequency as well (*p* = 0.004) and total score of the PSQI (*p* < 0.001). These two comparisons were still significant (*p* < 0.001) when using age as a covariate, as age is also a common factor to control in dream studies (Nielsen, 2012). As a consequence, every comparison made in the “Result” section will be controlled for gender and age. All of the variables in this study were compared between groups, this comparison is available in Supplementary Materials.

This study was carried out within the framework of French legislation on ethics and data protection. All participants completed a separate consent form that guaranteed anonymity, informed them of the scope of the study and the possibility of stopping it at any time.

### Material

Participants were requested to fill in a 150-question composite questionnaire online based on the study of Ribeiro et al. (2016). Only questions pertaining to this study are addressed in the following section.

Participants’ demographics and characteristics included gender and date of birth. The question concerning occupation concerned whether the participant was a student or not, and if they felt like their sleep schedule was constrained by their daily activity. The wording of this yes/no explorative question was “Do your professional, associative or domestic activities require you to go to bed or get up at specific times?”

In order to assess sleep quality, we used the PSQI total score which is based on 17 questions that evaluate seven components labeled as follows: sleep quality, sleep latency, sleep duration, sleep efficiency, sleep disturbances, medications, daytime dysfunction (Buysse et al., 1989; Léger et al., 2006). The PSQI is the most commonly used generic measure in clinical and research setting and has been demonstrated to have an adequate content validity, a good construct validity and a good discriminative validity (Mollayeva et al., 2016).

In the questionnaire, four questions on dream experiences concerned dream frequency, LD frequency, dreams with awareness frequency, and dreams with control frequency. These questions were reformulated in order to ensure a good comprehension in French language but were conceptually similar to those typically used in the literature (Stepansky et al., 1998; Watson, 2001; Fassler et al., 2006; Soffer-Dudek et al., 2011).

The wording of the dream frequency question was: “*In the past 6 months, how often have you been able to remember at least one of your dreams when you woke up?*” (0 = Less than once a month, 1 = Once a month, 2 = Two or three times a month, 3 = Once a week, 4 = Two or three times a week, and 5 = Four times a week or more).

The LD frequency question was preceded by a definition of LD: “*During* LD, *one is – while dreaming – aware of the fact that one is dreaming. It is possible to deliberately wake up or to control the dream action or to observe passively in the course of the dream with this awareness.*” The question was “*Referring to the definition below, how often have you experienced* LD*?*” (0 = never, 1 = less than once a year, 2 = about once a year, 3 = about 2–4 times a year, 4 = about once a month, 5 = about 2–3 times a month, 6 = about once a week, 7 = several times a week). The definition and frequency scale were extracted from Schredl and Erlacher (2004).

Awareness and control were both evaluated on the same 6-point rating scale (0 = never, 1 = once, 2 = Less than once a year but more than just once, 3 = many times a year, 4 = many times a month, 5 = many times a week). For awareness the wording of the question was “*While dreaming, have you ever been aware that you were actually dreaming?*” and to control the wording was “*While dreaming, have you ever been able to control the content of your dream?*”

The order of the questions concerning LD, awareness, and control was proposed in two versions (the question of LD was presented after or before the two questions on awareness and control). The original French wording for all questions are accessible as Supplementary Material of the present article.

### Procedure

By clicking on the hyperlink associated with the recruitment text, participants were redirected to the questionnaire hosted on a Google form. Once the questionnaire was completed, all responses were entered into an online spreadsheet and transferred to an Excel spreadsheet where duplicate data were excluded. During pretest, the estimated time for completing the questionnaire was 20 min or more. All statistics were performed using R and/or Jamovi (Fox and Weisberg, 2018; R Core Team, 2019; The jamovi project, 2020).

## Results

Results are presented aligned with our aims: description of the results, investigation of how dream experiences could be related to sleep characteristics as assessed by the PSQI, and sample comparison on each indicator of the present study.

All comparisons considered the effect of age and gender as these factors are known to potentially influence dream frequency (Schredl and Reinhard, 2008). All of the variables in this study were compared between groups, this comparison is available in Supplementary Materials.

### Descriptive Data on Dream Experience Frequencies and Sleep Characteristics

Summarized answers to questions about the frequency of dream experiences (frequency of dreaming, LD, consciousness, and control) are available in Table 1.

**TABLE 1:** Descriptive data for all dream-related experiences frequencies.

	Student *n* = 274		General pop. *n* = 681	
	Counts	Percentage	Counts	Percentage
**Dreaming**				
Less than once a month	23	8,39	163	23,94
Once a month	30	10,95	72	10,57
Two or three times a month	28	10,22	100	14,68
Once a week	55	20,07	117	17,18
Two or three times a week	83	30,29	143	21
Four or more times a week	55	20,07	86	12,63
**Lucid dreaming**				
Never	99	36,13	344	50,51
Less than once a year	43	15,69	80	11,75
About once a year	25	9,12	51	7,49
About 2 to 4 times a year	48	17,52	98	14,39
About once a month	25	9,12	43	6,31
About 2 to 3 times a month	17	6,2	36	5,29
About once a week	12	4,38	12	1,76
Several times a week	5	1,82	17	2,5
**Dream with awareness**				
Never	55	20,07	185	27,17
Once	25	9,12	70	10,28
Less than once a year	45	16,42	107	15,71
Many times a year	63	22,99	107	15,71
Many times a month	26	9,49	51	7,49
many times a week	60	21,9	161	23,64
**Dream with control**				
Never	391	44,53	122	57,42
Once	45	8,39	23	6,61
Less than once a year	83	13,5	37	12,19
Many times a year	55	14,6	40	8,08
Many times a month	25	4,01	11	3,67
Many times a week	82	14,96	41	12,04

Summarized answers to questions about sleep quality as assessed with the PSQI are summarized in Table 2.

**TABLE 2:** Descriptive data for the PSQI across the two groups.

	Student *n* = 274		General pop. *n* = 681	
	Counts	Percentage	Counts	Percentage
**Sleep quality**				
Score = 0	9	3,28	52	7,64
Score = 1	128	46,72	322	47,28
Score = 2	113	41,24	251	36,86
Score = 3	24	8,76	56	8,22
**Sleep latency**				
Score = 0	51	18,61	149	21,88
Score = 1	74	27,01	225	33,04
Score = 2	76	27,74	183	26,87
Score = 3	73	26,64	124	18,21
**Sleep duration**				
Score = 0	95	34,67	249	36,56
Score = 1	92	33,58	214	31,42
Score = 2	57	20,8	136	19,97
Score = 3	30	10,95	82	12,04
**Sleep efficiency**				
Score = 0	179	65,33	455	66,81
Score = 1	60	21,9	98	14,39
Score = 2	17	6,2	75	11,01
Score = 3	18	6,57	53	7,78
**Sleep disturbance**				
Score = 0	12	4,38	38	5,58
Score = 1	208	75,91	469	68,87
Score = 2	49	17,88	160	23,49
Score = 3	5	1,82	14	2,06
**Medication**				
Score = 0	240	87,59	593	87,08
Score = 1	9	3,28	33	4,85
Score = 2	13	4,74	14	2,06
Score = 3	12	4,38	41	6,02
**Daytime dysfunction**				
Score = 0	28	10,22	101	14,83
Score = 1	97	35,4	278	40,82
Score = 2	107	39,05	233	34,21
Score = 3	42	15,33	69	10,13
**PSQI Score, Mean (SD) *n* for score >5**	8.67 (3.41) *n* = 225		8.33 (3.46) *n* = 537	

### Sleep Quality and Dream Experience Frequencies, a Regression Analysis

We performed linear regression analysis to investigate whether the global PSQI score variance could be explained by LD frequency. Within this scope, dream experiences frequencies were recoded as a frequency per month using the class means. As indicated above, age and gender have been added as covariates and factors. Summary for this analysis is available in Table 3. Noticeably, the model was significant (with a *p*-value lower than 0.05) only when gender was added as a covariable; a closer analysis of the model coefficient confirmed that LD did not participate to this significance. In other world, lucid dream frequency does not help to predict sleep quality significantly. These tests were also performed for dream recall frequency, awareness, and control with similar outcomes suggesting that dream experience frequency does not predict significantly PSQI score.

**TABLE 3:** Model fit measures and model coefficient for the regression analyses concerning sleep quality and lucid dreaming frequency.

Model fit measures						
			Overall model test			
Model	*R*	*R*^2^	*F*	df1	df2	*p*
1 LDF_Recoded	0.0122	1.49e-4	0.142	1	953	0.706
LDF_Recoded						
2 LDF_Recoded and DRF_Recoded	0.0393	0.00154	0.735	2	952	0.480
LDF_Recoded						
3 LDF_Recoded, DRF_Recoded, and age	0.0561	0.00315	1.002	3	951	0.391
4 LDF_Recoded, DRF_Recoded, age, and college student	0.0711	0.00506	1.207	4	950	0.306
5 LDF_Recoded, DRF_Recoded, age, college student, and gender	0.1362	0.01854	3.585	5	949	0.003

*To isolate the singular effect of gender, we chose to test the prediction of the PSQI score by our variable of interest in five models by adding a control variable one by one. The model coefficients presented in the table beside concern the complete model (5) as being the only one significant. LDF: lucid dreaming frequency and DRF: Dream recall frequency.*						

**Model coefficients – PSQI_total.**						

**Predictor**	**Estimate**		**SE**	***t***	***p***	

Intercept	29.3085		17.13169	1.711	0.087	
LDF_Recoded	–0.0141		0.04014	–0.352	0.725	
DRF_Recoded	0.0128		0.01505	–0.352	0.725	
College_Student:			0.851	0.395	
Yes – No	0.3093		0.26823	1.153	0.249	
Birth	–0.0104		0.00864	–1.208	0.227	
Gender:				
Men – Women	(0.8753		0.24241	(3.611	(0.001	

Linear regression tables for the comparison mentioned above and for separate regression analysis depending on the group are available in the Supplementary Material.

## Discussion

This study was primarily conducted to evaluate how dream frequency could predict sleep quality in these two samples: a student and a general population sample. The research also aimed at describing the frequency of dream experiences (dreaming, LD, awareness, and control) and sleep quality as measured by the PSQI in these two samples.

Concerning LD frequency, individuals in the general population group have a prevalence (one or more occurrences during their lifetime) of LD of 49.49%. Even though the present general sample showed a wide age range, it was not a representative sample, however, dream recall frequency is close to that of a representative German sample in which 51% of participants reported having a lucid dream at least once (Schredl and Erlacher, 2011). In the same group, 15.86% were considered to have frequent lucid dreams because they had lucid dreams at least once a month, compared to 20.1% in the German representative sample (Snyder and Gackenbach, 1988). In the group of students, 63.87% reported having one or more lucid dreams, while 81.05% reported having such a dream in the 2015–2016 study (Ribeiro et al., 2016) and 82% of the student sample of Schadow et al. (2018). In the same group, 21.52% were frequent lucid dreamers, while 36.36% of students were considered lucid dreamers in 2015 and 36.9% in Schadow et al. (2018). In other words, the frequency of LD is lower in this study than in a previous study, while instructions and timing of data collection (beginning of the year) are noticeably similar (Ribeiro et al., 2016). This discrepancy could be explained by the fact that in 2015, students did not indicate which disciplines they were involved in, whereas the students in this study are all psychology students. Another explanation could come from the fact that participants saw all questions about consciousness and control in this questionnaire whereas they had only seen LD ones in the other study.

Concerning the answers to question about the dream of awareness and dream with control frequencies, participants were 72.83% to indicate one dream or more with awareness of the dream state in the general population group and 79.93% in the student group; they were 73.38% in the student group of the 2016 study (Ribeiro et al., 2016). Participants were 42.58% to indicate one dream or more with control of the dream state in the general population group and 55.47% of the student group; they were 50.65% in the student group of 2016 (Ribeiro et al., 2016).

This study did not find links between dream experiences frequencies (dream, lucid dream, awareness dreams, control dream) and sleep characteristics assessed with the PSQI for the student and the general group. Noticeably, the extent of the variance explained of significant models was rather low and only the gender predictor carried on this significance. Moreover, Schadow et al. (2018) have proposed that the occurrence of lucid dreams is not *per se* related to sleep quality but a consequence of higher nightmare frequencies. We believe that the present investigation participates in an accumulation of studies that invite to consider general LD occurrence as innocuous for sleep characteristics, but more studies are still needed. Aviram and Soffer-Dudek (2018) have indicated that LD can be beneficial or detrimental to a person’s well-being, depending on the context in which lucidity occurs, such as whether or not people have attempted to induce it. Some of these techniques can be expected to disrupt sleep parameters; for example, some dreamers use devices that randomly send a red light into the eye during sleep in the hope of waking the individual sufficiently to experience LD (Stumbrys et al., 2012; Mota-Rolim et al., 2019). Therefore, future investigation of relation between LD frequency and sleep quality should focus specifically on instances where there is an increase to its frequency (Vallat and Ruby, 2019; Soffer-Dudek, 2020). In light of the present study, we believe that these future studies would benefit from using several operational definitions of dream lucidity to conduct their investigations. The Frequency and Intensity Lucid Dreaming (FILD) questionnaire may be of interest in this regard (Aviram and Soffer-Dudek, 2018). Another proposition is the Lucid dreaming Skills Scale (LUSK) that investigates frequency of LD, awareness/perception, dream control, and problems associated with being lucid during dreams using 22 items (Schredl et al., 2018). As a comment on possible future study: it would also be important to assess whether individuals who use lucid dream induction methods do so in an attempt to cope with their sleep problem. Indeed, this simple fact could lead to a misinterpretation of LD as being detrimental to sleep quality (see discussion on Schredl et al., 2020).

Additionally, the state-of-the-art on typical dreams invites also to mitigate the proposition of a detrimental effect of dream recall frequency on sleep quality, for instance, a decline in sleep quality was associated with a decline in dream recall for individuals with insomnia (Pagel and Shocknesse, 2007). For future research, we recommend using the scale proposed in the MADRE questionnaire as its metric properties are better known as the one we used in this study (for a French validation see Schredl, 2004; Schredl et al., 2014; Ghorayeb et al., 2019).

## Conclusion

Frequencies of dream-related experiences were in the range of previous studies and 49.5% of individuals in the general population group indicated having experience LD at least once during their lifetime. No specific link was found between atypical dream consciousness frequencies and sleep quality as expressed with a total score of the PSQI. The present result and all the others that have failed to link LD to diminished sleep quality could be an invitation to conceptualize consciousness and control as phenomena that can participate in the diversity of dream phenomenology rather than as features of waking that are insinuated into dream phenomenology in a context of abnormality. Effect of induction strategies that impact directly sleep parameters on sleep quality remains to be investigated.

## Data Availability Statement

The dataset generated for this study is available in the Supplementary Material and on request to the corresponding author.

## Ethics Statement

Ethical review and approval was not required for the study on human participants in accordance with the local legislation and institutional requirements. The patients/participants provided their written informed consent to participate in this study.

## Author Contributions

NR conducted the study and wrote the manuscript. YG and VQ supervised the research and contributed to the writing. All authors contributed to the article and approved the submitted version.

## Conflict of Interest

The authors declare that the research was conducted in the absence of any commercial or financial relationships that could be construed as a potential conflict of interest.
