# Supplementary material for: Is There a Link Between Frequency of Dreams, Lucid Dreams, and Subjective Sleep Quality?
Source: Front Psychol. 2020 Jun 25;11:1290. doi: 10.3389/fpsyg.2020.01290 (PMC7330170; doi:10.3389/fpsyg.2020.01290)
Supplement: Supplementary file 1 [file Table_1.docx]

# Original (French) Wording in the questionnaire

| **Code in the Datafile** | **Wording in French** | **Answer possibilities** |
| --- | --- | --- |
| College_Student | Êtes-vous étudiant ? | Oui, Non |
| Gender | Vous êtes : | Un homme, une femme, autre |
| Birth | Année de naissance : | [Textbox] |
| Dream_Recall_Frequency | Au cours des six derniers mois, combien de fois avez-vous pu vous souvenir d'au moins un de vos rêves au réveil ? | 0 = Moins d'une fois par mois, 1 = Une fois par mois, 2 = Deux ou trois fois par mois, 3 = Une fois par semaine, 4 = Deux ou trois fois par semaine et 5 = Quatre fois par semaine ou plus |
| Awareness_Frequency | Tout en rêvant, avez-vous déjà eu conscience que vous rêviez ? | 0 = jamais, 1 = une fois, 2 = moins d'une fois par an mais plus d'une fois, 3 = plusieurs fois par an, 4 = plusieurs fois par mois, 5 = plusieurs fois par semaine |
| Control_Frequency | Tout en rêvant, avez-vous déjà été capable de contrôler le contenu de votre rêve ? | 0 = jamais, 1 = une fois, 2 = moins d'une fois par an mais plus d'une fois, 3 = plusieurs fois par an, 4 = plusieurs fois par mois, 5 = plusieurs fois par semaine |
| Lucid_Dreaming_Freqency | Lors d'un rêve lucide, on est - tout en rêvant - conscient du fait que l'on est en train de rêver. Il est possible de se réveiller délibérément ou de contrôler l'action du rêve ou d'observer passivement le déroulement du rêve avec cette conscience. En vous référent à la définition ci-dessus à quelle fréquence avez-vous expérimenté le rêve lucide | 0 = jamais, 1 = moins d'une fois par an, 2 = environ une fois par an, 3 = environ 2 à 4 fois par an, 4 = environ une fois par mois, 5 = environ 2 à 3 fois par mois, 6 = environ une fois par semaine, 7 = plusieurs fois par semaine |
| SleepScheduleContrainedByActivity | Vos activités professionnelles, associatives ou domestiques vous obligent à vous coucher ou à vous lever à des heures précises ? | Oui, Non |

For the PSQI Wording please refer to the Hotel Dieu sleep and Awareness center (Paris) version [retrieved](http://maxime.elbaz.free.fr/examens/psqi.pdf) 26/04/2020 – 22:30

Original (French) Wording in the questionnaire
