# Supplementary material for: Is There a Link Between Frequency of Dreams, Lucid Dreams, and Subjective Sleep Quality?
Source: Front Psychol. 2020 Jun 25;11:1290. doi: 10.3389/fpsyg.2020.01290 (PMC7330170; doi:10.3389/fpsyg.2020.01290)
Supplement: Supplementary file 2 [file Table_2.docx]

## Sample comparison

The two groups were compared using the T-test and the Mann-Whitney U (when there was a violation of the equality variance assumption). These comparisons were significant for the PSQI latency (*p* < .001; *d* = .20) and daytime dysfunction components (*p* < .001; *d* = .23), for the dream recall frequency (*p* < .001; *d* = .43), for the dream control frequency (*p* < .001; *d* = .22) and for the lucid dreaming frequency (*p* < .001; *d* = .22).

As a measure to control spurious results that would be due to age difference in dream experiences recall (see introduction), only participants from the general population with a date of birth comparable to ages of students (birth > 1993) were selected. In addition, in order to control a possible gender effect, the comparison was performed twice, one for women only and one for men only. For women, students (*n* = 214) compared to the general population (*n* = 236) no-show significant results were observed using the Man-Whitney U test. For men, comparison showed that students (*n* = 55) compared to the general population (*n* = 133) differed for the duration (*p* = .04) and daytime dysfunction (*p* = .008) components of the PSQI and for the PSQI total score (*p* = .044). Cohen’s d for these three comparisons were under 0.3 indicating a rather weak effect size.

**Independent Samples comparisons between student and general population**

| Independent Samples comparisons | | | | | | | | | | | | | |
| --- | --- | --- | --- | --- | --- | --- | --- | --- | --- | --- | --- | --- | --- |
|  |  |  |  |  |  |  |  |  |  |  |  |  |  |
| **Variable** | | **Test** | | **Statistic** | | **p** | | **Mean difference** | | **SE difference** | | **Cohen's d** | |
| **Age** |  | **Student's t** |  | **-13.4511** |  | **< .001** |  | **-12.83674** |  | **0.9543** |  | **-0.96230** |  |
| **Gender** |  | **χ²** |  | **21.4** |  | **< .001** |  |  |  |  |  |  |  |
| PSQI_Quality |  | Mann-Whitney U |  | 86981 |  | 0.074 |  | -1.39e−5 |  | 0.0528 |  | -0.13293 |  |
| **PSQI_Latence** |  | **Mann-Whitney U** |  | **82879** |  | **0.005** |  | **-4.58e−5** |  | **0.0741** |  | **-0.20263** |  |
| PSQI_Duration |  | Mann-Whitney U |  | 92548 |  | 0.839 |  | -5.88e−5 |  | 0.0725 |  | -0.00533 |  |
| PSQI_Efficiency |  | Mann-Whitney U |  | 92782 |  | 0.873 |  | 5.84e-5 |  | 0.0672 |  | 0.06118 |  |
| PSQI_Disturbance |  | Mann-Whitney U |  | 89041 |  | 0.165 |  | 7.39e-5 |  | 0.0397 |  | 0.08775 |  |
| PSQI_Medication |  | Mann-Whitney U |  | 92801 |  | 0.824 |  | 6.36e-5 |  | 0.0548 |  | 0.01445 |  |
| **PSQI_Daytime_Dysfonction** |  | **Mann-Whitney U** |  | **81729** |  | **0.001** |  | **-4.63e−5** |  | **0.0617** |  | **-0.23004** |  |
| PSQI_Total |  | Mann-Whitney U |  | 87354 |  | 0.130 |  | -5.49e−6 |  | 0.2450 |  | -0.09993 |  |
| **Dream_Recall_Frequency** |  | **Mann-Whitney U** |  | **70587** |  | **< .001** |  | **-1.000** |  | **0.1213** |  | **-0.43958** |  |
| Awareness_Frequency |  | Mann-Whitney U |  | 87061 |  | 0.099 |  | -4.98e−7 |  | 0.1338 |  | -0.11434 |  |
| **Control_Frequency** |  | **Mann-Whitney U** |  | **81014** |  | **< .001** |  | **-2.17e−5** |  | **0.1292** |  | **-0.22130** |  |
| **Lucid_Dreaming_Freqency** |  | **Mann-Whitney U** |  | **79766** |  | **< .001** |  | **-7.76e−6** |  | **0.1381** |  | **-0.22504** |  |

**Control Frequency comparison between student and general population and controlled for dream recall frequency**

| ANCOVA - Control_Recoded | | | | | | | | | | | |
| --- | --- | --- | --- | --- | --- | --- | --- | --- | --- | --- | --- |
|  |  |  |  |  |  |  |  |  |  |  |  |
|  | | **Sum of Squares** | | **df** | | **Mean Square** | | **F** | | **p** | |
| Overall model |  | 138.8 |  | 2 |  | 69.4 |  | 2.24 |  | 0.107 |  |
| College_Student |  | 37.0 |  | 1 |  | 37.0 |  | 1.03 |  | 0.311 |  |
| DRF_Recoded |  | 101.8 |  | 1 |  | 101.8 |  | 2.83 |  | 0.093 |  |
| Residuals |  | 34215.5 |  | 952 |  | 35.9 |  |  |  |  |  |
|  | | | | | | | | | | | |

**Lucid dreaming frequency comparison between student and general population and controlled for dream recall frequency**

| ANCOVA - LDF_Recoded | | | | | | | | | | | |
| --- | --- | --- | --- | --- | --- | --- | --- | --- | --- | --- | --- |
|  |  |  |  |  |  |  |  |  |  |  |  |
|  | | **Sum of Squares** | | **df** | | **Mean Square** | | **F** | | **p** | |
| **Overall model** |  | **134.30** |  | **2** |  | **67.15** |  | **8.804** |  | **< .001** |  |
| College_Student |  | 1.28 |  | 1 |  | 1.28 |  | 0.170 |  | 0.681 |  |
| **DRF_Recoded** |  | **133.01** |  | **1** |  | **133.01** |  | **17.554** |  | **< .001** |  |
| Residuals |  | 7213.56 |  | 952 |  | 7.58 |  |  |  |  |  |
|  | | | | | | | | | | | |

**Sample comparison for women with date of birth >1993 between college student (n = 214) and general population (n = 236)**

| Independent Samples T-Test | | | | | | | | | | | | | |
| --- | --- | --- | --- | --- | --- | --- | --- | --- | --- | --- | --- | --- | --- |
|  |  |  |  |  |  |  |  |  |  |  |  |  |  |
|  | |  | | **Statistic** | | **p** | | **Mean difference** | | **SE difference** | | **Cohen's d** | |
| Birth |  | Mann-Whitney U |  | 24771 |  | 0.719 |  | 3.89e-5 |  | 0.1560 |  | -0.11217 |  |
| PSQI_Quality |  | Mann-Whitney U |  | 24490 |  | 0.548 |  | -2.78e−5 |  | 0.0707 |  | -0.06025 |  |
| PSQI_Latence |  | Mann-Whitney U |  | 24595 |  | 0.621 |  | -8.33e−6 |  | 0.0979 |  | -0.03880 |  |
| PSQI_Duration |  | Mann-Whitney U |  | 23493 |  | 0.178 |  | -4.80e−5 |  | 0.0959 |  | -0.11530 |  |
| PSQI_Efficiency |  | Mann-Whitney U |  | 24782 |  | 0.686 |  | -1.62e−6 |  | 0.0880 |  | 6.80e-4 |  |
| PSQI_Disturbance |  | Mann-Whitney U |  | 25239 |  | 0.990 |  | 1.86e-5 |  | 0.0508 |  | 0.00375 |  |
| PSQI_Medication |  | Mann-Whitney U |  | 24629 |  | 0.437 |  | 9.21e-6 |  | 0.0735 |  | 0.08516 |  |
| PSQI_Daytime_Dysfonction |  | Mann-Whitney U |  | 23805 |  | 0.265 |  | -4.11e−5 |  | 0.0805 |  | -0.11054 |  |
| PSQI_Total |  | Student's t |  | -0.75893 |  | 0.448 |  | -1.56e−5 |  | 0.3298 |  | -0.07164 |  |
| Dream_Recall_Frequency |  | Mann-Whitney U |  | 25173 |  | 0.953 |  | -1.40e−5 |  | 0.1448 |  | -0.01200 |  |
| Awareness_Frequency |  | Mann-Whitney U |  | 24841 |  | 0.761 |  | 5.19e-5 |  | 0.1735 |  | 0.02790 |  |
| Control_Frequency |  | Mann-Whitney U |  | 25012 |  | 0.853 |  | 4.88e-5 |  | 0.1817 |  | 0.02685 |  |
| Lucid_Dreaming_Frequency |  | Mann-Whitney U |  | 25228 |  | 0.986 |  | 4.68e-5 |  | 0.1918 |  | 0.00974 |  |
|  | | | | | | | | | | | | | |

**Sample comparison for men with date of birth >1993 between college student (n = 55) and general population (n = 133)**

| Independent Samples T-Test | | | | | | | | | | | | | |
| --- | --- | --- | --- | --- | --- | --- | --- | --- | --- | --- | --- | --- | --- |
|  |  |  |  |  |  |  |  |  |  |  |  |  |  |
|  | |  | | **Statistic** | | **p** | | **Mean difference** | | **SE difference** | | **Cohen's d** | |
| Birth |  | Mann-Whitney U |  | 47161 |  | 0.271 |  | -2.60e−5 |  | 0.2963 |  | -0.1931 |  |
| PSQI_Quality |  | Mann-Whitney U |  | 46892 |  | 0.195 |  | -4.29e−5 |  | 0.1183 |  | -0.1194 |  |
| PSQI_Latence |  | Mann-Whitney U |  | 48170 |  | 0.510 |  | -3.26e−5 |  | 0.1639 |  | -0.0481 |  |
| **PSQI_Duration** |  | **Mann-Whitney U** |  | **45099** |  | **0.037** |  | **-3.79e−5** |  | **0.1523** |  | **-0.1525** |  |
| PSQI_Efficiency |  | Mann-Whitney U |  | 47118 |  | 0.180 |  | -6.65e−5 |  | 0.1135 |  | -0.0541 |  |
| PSQI_Disturbance |  | Mann-Whitney U |  | 48652 |  | 0.573 |  | -3.50e−5 |  | 0.0793 |  | -0.0434 |  |
| PSQI_Medication |  | Mann-Whitney U |  | 48955 |  | 0.595 |  | -6.85e−5 |  | 0.0897 |  | -0.0309 |  |
| **PSQI_Daytime_Dysfonction** |  | **Mann-Whitney U** |  | **43836** |  | **0.008** |  | **-7.27e−6** |  | **0.1401** |  | **-0.2173** |  |
| **PSQI_Total** |  | **Student's t** |  | **-1.7017** |  | **0.044** |  | **-0.83295** |  | **0.4895** |  | **-0.27281** |  |
| Dream_Recall_Frequency |  | Mann-Whitney U |  | 47741 |  | 0.401 |  | -1.65e−5 |  | 0.2673 |  | -0.0782 |  |
| Awareness_Frequency |  | Mann-Whitney U |  | 49106 |  | 0.816 |  | -2.56e−5 |  | 0.2881 |  | -0.0223 |  |
| Control_Frequency |  | Mann-Whitney U |  | 48398 |  | 0.569 |  | -5.83e−5 |  | 0.2863 |  | -0.0275 |  |
| Lucid_Dreaming_Frequency |  | Mann-Whitney U |  | 46738 |  | 0.190 |  | -6.93e−5 |  | 0.3223 |  | -0.0681 |  |
|  | | | | | | | | | | | | | |

The jamovi project (2020). *jamovi*. (Version 1.2) [Computer Software]. Retrieved from <https://www.jamovi.org>.

R Core Team (2019). *R: A Language and environment for statistical computing*. (Version 3.6) [Computer software]. Retrieved from <https://cran.r-project.org/>.

Fox, J., & Weisberg, S. (2018). *car: Companion to Applied Regression*. [R package]. Retrieved from <https://cran.r-project.org/package=car>.

Garbett, S. (2018). *tangram: The Grammar of Tables*. [R package]. Retrieved from <https://CRAN.R-project.org/package=tangram>.
