# Supplementary material for: Is There a Link Between Frequency of Dreams, Lucid Dreams, and Subjective Sleep Quality?
Source: Front Psychol. 2020 Jun 25;11:1290. doi: 10.3389/fpsyg.2020.01290 (PMC7330170; doi:10.3389/fpsyg.2020.01290)
Supplement: Supplementary file 3 [file Table_3.docx]

## Gender, age, and occupation comparison

In study investigating subjective sleep parameters, the gender can rationally be supposed to influence the results (see Schredl & Reinhard, 2008). Dream experiences frequencies and score of the PSQI have been compared across genders. In the student group, comparison was significant for dream recall frequencies (*p* = .018). In the general population, the comparison was significant for dream recall frequency as well (*p =* .004) and total score of the PSQI (*p <* .001). These two comparisons were still significant (*p* < .001) when using age as a covariate. As a consequence, every comparison made in the result section was controlled for gender and age.

Concerning the explorative questions about their occupation, only sleep duration components of the PSQI in the general population group was different depending on the answer. Namely, the Mann-Whitney U revealed that participants who indicated having activities that required them to go to bed and wake at specific hours (n = 317 over 681 participants in this group) had a higher duration score on the PSQI scale (M = 1.23 SD = 1.06) than participants who did not (M = 0.94, SD = 0.965) (*p* < .001)

**Independent Samples comparisons between genders in the student population**

| Independent Samples T-Test | | | | | | | | | | | | | |
| --- | --- | --- | --- | --- | --- | --- | --- | --- | --- | --- | --- | --- | --- |
|  |  |  |  |  |  |  |  |  |  |  |  |  |  |
|  | |  | | **Statistic** | | **p** | | **Mean difference** | | **SE difference** | | **Cohen's d** | |
| Birth |  | Mann-Whitney U |  | 5594 |  | 0.398 |  | -7.39e−5 |  |  |  | 0.07560 |  |
| PSQI_Quality |  | Mann-Whitney U |  | 5910 |  | 0.814 |  | -1.31e−5 |  |  |  | -0.01658 |  |
| PSQI_Latence |  | Mann-Whitney U |  | 5834 |  | 0.711 |  | -1.47e−5 |  |  |  | -0.04937 |  |
| PSQI_Duration |  | Mann-Whitney U |  | 6012 |  | 0.984 |  | -3.21e−5 |  |  |  | -0.03232 |  |
| PSQI_Efficiency |  | Mann-Whitney U |  | 5336 |  | 0.121 |  | -1.18e−5 |  |  |  | -0.27892 |  |
| PSQI_Disturbance |  | Mann-Whitney U |  | 5327 |  | 0.076 |  | -4.31e−5 |  |  |  | -0.28450 |  |
| PSQI_Medication |  | Mann-Whitney U |  | 5869 |  | 0.611 |  | 3.51e-5 |  |  |  | 0.05346 |  |
| PSQI_Daytime_Dysfonction |  | Mann-Whitney U |  | 5900 |  | 0.806 |  | 2.54e-5 |  |  |  | 0.00735 |  |
| **Dream_Recall_Frequency** |  | **Mann-Whitney U** |  | **4797** |  | **0.017** |  | **-1.000** |  |  |  | **-0.35930** |  |
| Awareness_Frequency |  | Mann-Whitney U |  | 5664 |  | 0.487 |  | 2.18e-7 |  |  |  | 0.12671 |  |
| Control_Frequency |  | Mann-Whitney U |  | 5687 |  | 0.501 |  | 4.56e-5 |  |  |  | 0.04236 |  |
| Lucid_Dreaming_Frequency |  | Mann-Whitney U |  | 5378 |  | 0.206 |  | 2.02e-5 |  |  |  | 0.12502 |  |
| PSQI_Total |  | Mann-Whitney U |  | 5734 |  | 0.582 |  | -5.51e−5 |  |  |  | -0.10610 |  |
|  | | | | | | | | | | | | | |

| ANCOVA - DRF_Recoded | | | | | | | | | | | |
| --- | --- | --- | --- | --- | --- | --- | --- | --- | --- | --- | --- |
|  |  |  |  |  |  |  |  |  |  |  |  |
|  | | **Sum of Squares** | | **df** | | **Mean Square** | | **F** | | **p** | |
| Overall model |  | 503 |  | 2 |  | 251.6 |  | 3.67 |  | 0.027 |  |
| Gender |  | 380 |  | 1 |  | 380.2 |  | 5.69 |  | 0.018 |  |
| Birth |  | 123 |  | 1 |  | 123.0 |  | 1.84 |  | 0.176 |  |
| Residuals |  | 18106 |  | 271 |  | 66.8 |  |  |  |  |  |
|  | | | | | | | | | | | |

**Independent Samples comparisons between genders in the general population**

| Independent Samples T-Test | | | | | | | | | | | | | |
| --- | --- | --- | --- | --- | --- | --- | --- | --- | --- | --- | --- | --- | --- |
|  |  |  |  |  |  |  |  |  |  |  |  |  |  |
|  | |  | | **Statistic** | | **p** | | **Mean difference** | | **SE difference** | | **Cohen's d** | |
| Birth |  | Mann-Whitney U |  | 49998 |  | 0.217 |  | -1.000 |  |  |  | 0.01596 |  |
| PSQI_Quality |  | Mann-Whitney U |  | 49517 |  | 0.120 |  | -5.86e−5 |  |  |  | -0.13727 |  |
| PSQI_Latence |  | Mann-Whitney U |  | 50695 |  | 0.326 |  | -8.96e−5 |  |  |  | -0.08036 |  |
| PSQI_Duration |  | Mann-Whitney U |  | 52488 |  | 0.820 |  | -8.78e−5 |  |  |  | -0.03801 |  |
| **PSQI_Efficiency** |  | **Mann-Whitney U** |  | **46335** |  | **0.001** |  | **-3.07e−5** |  |  |  | **-0.26192** |  |
| **PSQI_Disturbance** |  | **Mann-Whitney U** |  | **45802** |  | **< .001** |  | **-1.45e−5** |  |  |  | **-0.30821** |  |
| **PSQI_Medication** |  | **Mann-Whitney U** |  | **50094** |  | **0.041** |  | **-1.01e−5** |  |  |  | **-0.19225** |  |
| **PSQI_Daytime_Dysfonction** |  | **Mann-Whitney U** |  | **46192** |  | **0.003** |  | **-4.68e−7** |  |  |  | **-0.25217** |  |
| **Dream_Recall_Frequency** |  | **Mann-Whitney U** |  | **46001** |  | **0.004** |  | **-8.91e−6** |  |  |  | **-0.23256** |  |
| Awareness_Frequency |  | Mann-Whitney U |  | 51204 |  | 0.450 |  | -4.99e−5 |  |  |  | -0.05779 |  |
| Control_Frequency |  | Mann-Whitney U |  | 51114 |  | 0.387 |  | -3.76e−5 |  |  |  | -0.06308 |  |
| Lucid_Dreaming_Frequency |  | Mann-Whitney U |  | 51749 |  | 0.578 |  | -1.79e−5 |  |  |  | -0.00206 |  |
| **PSQI_Total** |  | **Mann-Whitney U** |  | **44569** |  | **< .001** |  | **-1.000** |  |  |  | **-0.31142** |  |
|  | | | | | | | | | | | | | |

**How Age and Gender predict dream recall frequency in the general population group using an Ordinal Logistic Regression**

| \| Model Fit Measures \| \| \| \| \| \| \| \| \| \| \| \| \| \| \| --- \| --- \| --- \| --- \| --- \| --- \| --- \| --- \| --- \| --- \| --- \| --- \| --- \| --- \| \|  \| \| \| \| \| \| \| \| **Overall Model Test** \| \| \| \| \| \| \| **Model** \| \| **Deviance** \| \| **AIC** \| \| **R²_McF_** \| \| **χ²** \| \| **df** \| \| **p** \| \| \| 1 \|  \| 918 \|  \| 932 \|  \| 0.00805 \|  \| 7.45 \|  \| 2 \|  \| 0.024 \|  \| \| Note. The dependent variable 'Dream_Recall_Frequency' has the following order: 0 \| 1 \| 2 \| 3 \| 4 \| 5 \| \| \| \| \| \| \| \| \| \| \| \| \| \| \|  \| \| \| \| \| \| \| \| \| \| \| \| \| \| | \| Model Coefficients - Dream_Recall_Frequency \| \| \| \| \| \| \| \| \| \| \| --- \| --- \| --- \| --- \| --- \| --- \| --- \| --- \| --- \| --- \| \|  \|  \|  \|  \|  \|  \|  \|  \|  \|  \| \| **Predictor** \| \| **Estimate** \| \| **SE** \| \| **Z** \| \| **p** \| \| \| Birth \|  \| 0.0407 \|  \| 1.53e-4 \|  \| 265.25 \|  \| < .001 \|  \| \| Gender: \|  \|  \|  \|  \|  \|  \|  \|  \|  \| \| Women – Men \|  \| 0.6487 \|  \| 0.270 \|  \| 2.40 \|  \| 0.016 \|  \| \|  \| \| \| \| \| \| \| \| \| \| |
| --- | --- | --- | --- | --- | --- | --- | --- | --- | --- | --- | --- | --- | --- | --- | --- | --- | --- | --- | --- | --- | --- | --- | --- | --- | --- | --- | --- | --- | --- | --- | --- | --- | --- | --- | --- | --- | --- | --- | --- | --- | --- | --- | --- | --- | --- | --- | --- | --- | --- | --- | --- | --- | --- | --- | --- | --- | --- | --- | --- | --- | --- | --- | --- | --- | --- | --- | --- | --- | --- | --- | --- | --- | --- | --- | --- | --- | --- | --- | --- | --- | --- | --- | --- | --- | --- | --- | --- | --- | --- | --- | --- | --- | --- | --- | --- | --- | --- | --- | --- | --- | --- | --- | --- | --- | --- | --- | --- | --- | --- | --- | --- | --- | --- | --- | --- | --- | --- | --- | --- | --- | --- | --- | --- | --- | --- | --- | --- | --- | --- | --- | --- | --- | --- | --- | --- | --- | --- | --- | --- | --- | --- | --- | --- | --- | --- | --- | --- | --- | --- | --- | --- | --- | --- | --- | --- |

**How Age and Gender predict dream recall frequency in the general population group using an Ordinal Logistic Regression**

| \| Model Fit Measures \| \| \| \| \| \| \| \| \| \| \| \| \| \| \| --- \| --- \| --- \| --- \| --- \| --- \| --- \| --- \| --- \| --- \| --- \| --- \| --- \| --- \| \|  \| \| \| \| \| \| \| \| **Overall Model Test** \| \| \| \| \| \| \| **Model** \| \| **Deviance** \| \| **AIC** \| \| **R²_McF_** \| \| **χ²** \| \| **df** \| \| **p** \| \| \| 1 \|  \| 918 \|  \| 932 \|  \| 0.00805 \|  \| 7.45 \|  \| 2 \|  \| 0.024 \|  \| \| Note. The dependent variable 'Dream_Recall_Frequency' has the following order: 0 \| 1 \| 2 \| 3 \| 4 \| 5 \| \| \| \| \| \| \| \| \| \| \| \| \| \| \|  \| \| \| \| \| \| \| \| \| \| \| \| \| \| | \| Model Coefficients - Dream_Recall_Frequency \| \| \| \| \| \| \| \| \| \| \| --- \| --- \| --- \| --- \| --- \| --- \| --- \| --- \| --- \| --- \| \|  \|  \|  \|  \|  \|  \|  \|  \|  \|  \| \| **Predictor** \| \| **Estimate** \| \| **SE** \| \| **Z** \| \| **p** \| \| \| Birth \|  \| 0.0407 \|  \| 1.53e-4 \|  \| 265.25 \|  \| < .001 \|  \| \| Gender: \|  \|  \|  \|  \|  \|  \|  \|  \|  \| \| Women – Men \|  \| 0.6487 \|  \| 0.270 \|  \| 2.40 \|  \| 0.016 \|  \| \|  \| \| \| \| \| \| \| \| \| \| |
| --- | --- | --- | --- | --- | --- | --- | --- | --- | --- | --- | --- | --- | --- | --- | --- | --- | --- | --- | --- | --- | --- | --- | --- | --- | --- | --- | --- | --- | --- | --- | --- | --- | --- | --- | --- | --- | --- | --- | --- | --- | --- | --- | --- | --- | --- | --- | --- | --- | --- | --- | --- | --- | --- | --- | --- | --- | --- | --- | --- | --- | --- | --- | --- | --- | --- | --- | --- | --- | --- | --- | --- | --- | --- | --- | --- | --- | --- | --- | --- | --- | --- | --- | --- | --- | --- | --- | --- | --- | --- | --- | --- | --- | --- | --- | --- | --- | --- | --- | --- | --- | --- | --- | --- | --- | --- | --- | --- | --- | --- | --- | --- | --- | --- | --- | --- | --- | --- | --- | --- | --- | --- | --- | --- | --- | --- | --- | --- | --- | --- | --- | --- | --- | --- | --- | --- | --- | --- | --- | --- | --- | --- | --- | --- | --- | --- | --- | --- | --- | --- | --- | --- | --- | --- | --- | --- |

**Does PSQI Total score is different across gender when controlling for age using an ANCOVA**

| ANCOVA - PSQI_Total | | | | | | | | | | | |
| --- | --- | --- | --- | --- | --- | --- | --- | --- | --- | --- | --- |
|  |  |  |  |  |  |  |  |  |  |  |  |
|  | | **Sum of Squares** | | **df** | | **Mean Square** | | **F** | | **p** | |
| Gender |  | 173.2 |  | 1 |  | 173.2 |  | 15.06 |  | < .001 |  |
| Birth |  | 18.5 |  | 1 |  | 18.5 |  | 1.61 |  | 0.205 |  |
| Residuals |  | 7786.0 |  | 677 |  | 11.5 |  |  |  |  |  |
|  | | | | | | | | | | | |

**How Age and Gender predict PSQI (Efficiency) in the general population group using a linear Regression**

| \| Model Fit Measures \| \| \| \| \| \| \| \| \| \| \| \| \| \| \| --- \| --- \| --- \| --- \| --- \| --- \| --- \| --- \| --- \| --- \| --- \| --- \| --- \| --- \| \|  \| \| \| \| \| \| **Overall Model Test** \| \| \| \| \| \| \| \| \| **Model** \| \| **R** \| \| **R²** \| \| **F** \| \| **df1** \| \| **df2** \| \| **p** \| \| \| 1 \|  \| 0.184 \|  \| 0.0339 \|  \| 11.9 \|  \| 2 \|  \| 678 \|  \| < .001 \|  \| \|  \| \| \| \| \| \| \| \| \| \| \| \| \| \| | \| Model Coefficients - PSQI_Efficiency \| \| \| \| \| \| \| \| \| \| \| --- \| --- \| --- \| --- \| --- \| --- \| --- \| --- \| --- \| --- \| \|  \|  \|  \|  \|  \|  \|  \|  \|  \|  \| \| **Predictor** \| \| **Estimate** \| \| **SE** \| \| **t** \| \| **p** \| \| \| Intercept ᵃ \|  \| 17.05055 \|  \| 4.61907 \|  \| 3.69 \|  \| < .001 \|  \| \| Gender: \|  \|  \|  \|  \|  \|  \|  \|  \|  \| \| Women – Men \|  \| 0.24859 \|  \| 0.07604 \|  \| 3.27 \|  \| 0.001 \|  \| \| Birth \|  \| -0.00837 \|  \| 0.00233 \|  \| -3.60 \|  \| < .001 \|  \| \| ᵃ Represents reference level \| \| \| \| \| \| \| \| \| \| \|  \| \| \| \| \| \| \| \| \| \| |
| --- | --- | --- | --- | --- | --- | --- | --- | --- | --- | --- | --- | --- | --- | --- | --- | --- | --- | --- | --- | --- | --- | --- | --- | --- | --- | --- | --- | --- | --- | --- | --- | --- | --- | --- | --- | --- | --- | --- | --- | --- | --- | --- | --- | --- | --- | --- | --- | --- | --- | --- | --- | --- | --- | --- | --- | --- | --- | --- | --- | --- | --- | --- | --- | --- | --- | --- | --- | --- | --- | --- | --- | --- | --- | --- | --- | --- | --- | --- | --- | --- | --- | --- | --- | --- | --- | --- | --- | --- | --- | --- | --- | --- | --- | --- | --- | --- | --- | --- | --- | --- | --- | --- | --- | --- | --- | --- | --- | --- | --- | --- | --- | --- | --- | --- | --- | --- | --- | --- | --- | --- | --- | --- | --- | --- | --- | --- | --- | --- | --- | --- | --- | --- | --- | --- | --- | --- | --- | --- | --- | --- | --- | --- | --- | --- | --- | --- | --- | --- | --- | --- | --- | --- | --- | --- | --- | --- | --- | --- | --- | --- | --- |

**How Age and Gender predict PSQI (Disturbance) in the general population group using a linear Regression**

| \| Model Fit Measures \| \| \| \| \| \| \| \| \| \| \| \| \| \| \| --- \| --- \| --- \| --- \| --- \| --- \| --- \| --- \| --- \| --- \| --- \| --- \| --- \| --- \| \|  \| \| \| \| \| \| **Overall Model Test** \| \| \| \| \| \| \| \| \| **Model** \| \| **R** \| \| **R²** \| \| **F** \| \| **df1** \| \| **df2** \| \| **p** \| \| \| 1 \|  \| 0.203 \|  \| 0.0413 \|  \| 14.6 \|  \| 2 \|  \| 678 \|  \| < .001 \|  \| \|  \| \| \| \| \| \| \| \| \| \| \| \| \| \| | \| Model Coefficients - PSQI_Disturbance \| \| \| \| \| \| \| \| \| \| \| --- \| --- \| --- \| --- \| --- \| --- \| --- \| --- \| --- \| --- \| \|  \|  \|  \|  \|  \|  \|  \|  \|  \|  \| \| **Predictor** \| \| **Estimate** \| \| **SE** \| \| **t** \| \| **p** \| \| \| Intercept ᵃ \|  \| 11.33402 \|  \| 2.72129 \|  \| 4.16 \|  \| < .001 \|  \| \| Gender: \|  \|  \|  \|  \|  \|  \|  \|  \|  \| \| Women – Men \|  \| 0.17265 \|  \| 0.04480 \|  \| 3.85 \|  \| < .001 \|  \| \| Birth \|  \| -0.00515 \|  \| 0.00137 \|  \| -3.76 \|  \| < .001 \|  \| \| ᵃ Represents reference level \| \| \| \| \| \| \| \| \| \| \|  \| \| \| \| \| \| \| \| \| \| |
| --- | --- | --- | --- | --- | --- | --- | --- | --- | --- | --- | --- | --- | --- | --- | --- | --- | --- | --- | --- | --- | --- | --- | --- | --- | --- | --- | --- | --- | --- | --- | --- | --- | --- | --- | --- | --- | --- | --- | --- | --- | --- | --- | --- | --- | --- | --- | --- | --- | --- | --- | --- | --- | --- | --- | --- | --- | --- | --- | --- | --- | --- | --- | --- | --- | --- | --- | --- | --- | --- | --- | --- | --- | --- | --- | --- | --- | --- | --- | --- | --- | --- | --- | --- | --- | --- | --- | --- | --- | --- | --- | --- | --- | --- | --- | --- | --- | --- | --- | --- | --- | --- | --- | --- | --- | --- | --- | --- | --- | --- | --- | --- | --- | --- | --- | --- | --- | --- | --- | --- | --- | --- | --- | --- | --- | --- | --- | --- | --- | --- | --- | --- | --- | --- | --- | --- | --- | --- | --- | --- | --- | --- | --- | --- | --- | --- | --- | --- | --- | --- | --- | --- | --- | --- | --- | --- | --- | --- | --- | --- | --- | --- |

**How Age and Gender predict PSQI (Medication) in the general population group using a linear Regression**

| \| Model Fit Measures \| \| \| \| \| \| \| \| \| \| \| \| \| \| \| --- \| --- \| --- \| --- \| --- \| --- \| --- \| --- \| --- \| --- \| --- \| --- \| --- \| --- \| \|  \| \| \| \| \| \| **Overall Model Test** \| \| \| \| \| \| \| \| \| **Model** \| \| **R** \| \| **R²** \| \| **F** \| \| **df1** \| \| **df2** \| \| **p** \| \| \| 1 \|  \| 0.106 \|  \| 0.0112 \|  \| 3.84 \|  \| 2 \|  \| 678 \|  \| 0.022 \|  \| \|  \| \| \| \| \| \| \| \| \| \| \| \| \| \| | \| Model Coefficients - PSQI_Medication \| \| \| \| \| \| \| \| \| \| \| --- \| --- \| --- \| --- \| --- \| --- \| --- \| --- \| --- \| --- \| \|  \|  \|  \|  \|  \|  \|  \|  \|  \|  \| \| **Predictor** \| \| **Estimate** \| \| **SE** \| \| **t** \| \| **p** \| \| \| Intercept ᵃ \|  \| 5.38430 \|  \| 3.75672 \|  \| 1.43 \|  \| 0.152 \|  \| \| Gender: \|  \|  \|  \|  \|  \|  \|  \|  \|  \| \| Women – Men \|  \| 0.14780 \|  \| 0.06184 \|  \| 2.39 \|  \| 0.017 \|  \| \| Birth \|  \| -0.00262 \|  \| 0.00189 \|  \| -1.39 \|  \| 0.166 \|  \| \| ᵃ Represents reference level \| \| \| \| \| \| \| \| \| \| \|  \| \| \| \| \| \| \| \| \| \| |
| --- | --- | --- | --- | --- | --- | --- | --- | --- | --- | --- | --- | --- | --- | --- | --- | --- | --- | --- | --- | --- | --- | --- | --- | --- | --- | --- | --- | --- | --- | --- | --- | --- | --- | --- | --- | --- | --- | --- | --- | --- | --- | --- | --- | --- | --- | --- | --- | --- | --- | --- | --- | --- | --- | --- | --- | --- | --- | --- | --- | --- | --- | --- | --- | --- | --- | --- | --- | --- | --- | --- | --- | --- | --- | --- | --- | --- | --- | --- | --- | --- | --- | --- | --- | --- | --- | --- | --- | --- | --- | --- | --- | --- | --- | --- | --- | --- | --- | --- | --- | --- | --- | --- | --- | --- | --- | --- | --- | --- | --- | --- | --- | --- | --- | --- | --- | --- | --- | --- | --- | --- | --- | --- | --- | --- | --- | --- | --- | --- | --- | --- | --- | --- | --- | --- | --- | --- | --- | --- | --- | --- | --- | --- | --- | --- | --- | --- | --- | --- | --- | --- | --- | --- | --- | --- | --- | --- | --- | --- | --- | --- | --- |

**How Age and Gender predict PSQI (Efficiency) in the general population group using a linear Regression**

| \| Model Fit Measures \| \| \| \| \| \| \| \| \| \| \| \| \| \| \| --- \| --- \| --- \| --- \| --- \| --- \| --- \| --- \| --- \| --- \| --- \| --- \| --- \| --- \| \|  \| \| \| \| \| \| **Overall Model Test** \| \| \| \| \| \| \| \| \| **Model** \| \| **R** \| \| **R²** \| \| **F** \| \| **df1** \| \| **df2** \| \| **p** \| \| \| 1 \|  \| 0.122 \|  \| 0.0150 \|  \| 5.15 \|  \| 2 \|  \| 678 \|  \| 0.006 \|  \| \|  \| \| \| \| \| \| \| \| \| \| \| \| \| \| | \| Model Coefficients - PSQI_Daytime_Dysfonction \| \| \| \| \| \| \| \| \| \| \| --- \| --- \| --- \| --- \| --- \| --- \| --- \| --- \| --- \| --- \| \|  \|  \|  \|  \|  \|  \|  \|  \|  \|  \| \| **Predictor** \| \| **Estimate** \| \| **SE** \| \| **t** \| \| **p** \| \| \| Intercept ᵃ \|  \| -1.39733 \|  \| 4.16163 \|  \| -0.336 \|  \| 0.737 \|  \| \| Gender: \|  \|  \|  \|  \|  \|  \|  \|  \|  \| \| Women – Men \|  \| 0.21580 \|  \| 0.06851 \|  \| 3.150 \|  \| 0.002 \|  \| \| Birth \|  \| 0.00134 \|  \| 0.00210 \|  \| 0.638 \|  \| 0.524 \|  \| \| ᵃ Represents reference level \| \| \| \| \| \| \| \| \| \| \|  \| \| \| \| \| \| \| \| \| \| |
| --- | --- | --- | --- | --- | --- | --- | --- | --- | --- | --- | --- | --- | --- | --- | --- | --- | --- | --- | --- | --- | --- | --- | --- | --- | --- | --- | --- | --- | --- | --- | --- | --- | --- | --- | --- | --- | --- | --- | --- | --- | --- | --- | --- | --- | --- | --- | --- | --- | --- | --- | --- | --- | --- | --- | --- | --- | --- | --- | --- | --- | --- | --- | --- | --- | --- | --- | --- | --- | --- | --- | --- | --- | --- | --- | --- | --- | --- | --- | --- | --- | --- | --- | --- | --- | --- | --- | --- | --- | --- | --- | --- | --- | --- | --- | --- | --- | --- | --- | --- | --- | --- | --- | --- | --- | --- | --- | --- | --- | --- | --- | --- | --- | --- | --- | --- | --- | --- | --- | --- | --- | --- | --- | --- | --- | --- | --- | --- | --- | --- | --- | --- | --- | --- | --- | --- | --- | --- | --- | --- | --- | --- | --- | --- | --- | --- | --- | --- | --- | --- | --- | --- | --- | --- | --- | --- | --- | --- | --- | --- | --- | --- |

The jamovi project (2020). *jamovi*. (Version 1.2) [Computer Software]. Retrieved from <https://www.jamovi.org>.

R Core Team (2019). *R: A Language and environment for statistical computing*. (Version 3.6) [Computer software]. Retrieved from <https://cran.r-project.org/>.

Fox, J., & Weisberg, S. (2018). *car: Companion to Applied Regression*. [R package]. Retrieved from <https://cran.r-project.org/package=car>.

Garbett, S. (2018). *tangram: The Grammar of Tables*. [R package]. Retrieved from <https://CRAN.R-project.org/package=tangram>.
