# Supplementary material for: Is There a Link Between Frequency of Dreams, Lucid Dreams, and Subjective Sleep Quality?
Source: Front Psychol. 2020 Jun 25;11:1290. doi: 10.3389/fpsyg.2020.01290 (PMC7330170; doi:10.3389/fpsyg.2020.01290)
Supplement: Supplementary file 4 [file Table_4.docx]

**Regression - Awareness Frequency**

| Model Fit Measures | | | | | | | | | | | | | | | | | | | | | |  |  |
| --- | --- | --- | --- | --- | --- | --- | --- | --- | --- | --- | --- | --- | --- | --- | --- | --- | --- | --- | --- | --- | --- | --- | --- |
|  | | | | | | | | **Overall Model Test** | | | | | | | | | | | | | |  |  |
| **Model** | | **R** | | **R²** | | | | **F** | | | | **df1** | | | **df2** | | | **p** | | | |  |  |
| 1 | Awareness_Recoded | 0.0263 |  | 6.90e-4 | | |  | 0.658 | | |  | 1 |  | | 953 | |  | 0.417 | | |  |  |  |
| 2 | Awareness_Recoded DRF_Recoded | 0.0441 |  | 0.00194 | | |  | 0.925 | | |  | 2 |  | | 952 | |  | 0.397 | | |  |  |  |
| 3 | Awareness_Recoded DRF_Recoded  Age | 0.0595 |  | 0.00354 | | |  | 1.125 | | |  | 3 |  | | 951 | |  | 0.338 | | |  |  |  |
| 4 | Awareness_Recoded DRF_Recoded  Age  College_Student: | 0.0745 |  | 0.00555 | | |  | 1.324 | | |  | 4 |  | | 950 | |  | 0.259 | | |  |  |  |
| 5 | Awareness_Recoded DRF_Recoded  Age  College_Student  Gender | 0.1387 |  | 0.01923 | | |  | 3.721 | | |  | 5 |  | | 949 | |  | 0.002 | | |  |  |  |
|  | | | | | | | | | | | | | | | | | | | | | |  |  |
| Model Coefficients - PSQI_Total | | | | | | | | | | | | | | | | | | | | | | | |
|  | | | | |  |  | | |  |  | | | |  | |  | | |  |  | | |  |
| **Predictor** | | | | | | **Estimate** | | | | **SE** | | | | | | **t** | | | | **p** | | | |
| Intercept ᵃ | | | | |  | 29.8452 | | |  | 17.09040 | | | |  | | 1.746 | | |  | 0.081 | | |  |
| Awareness_Recoded | | | | |  | -0.0131 | | |  | 0.01471 | | | |  | | -0.889 | | |  | 0.374 | | |  |
| DRF_Recoded | | | | |  | 0.0123 | | |  | 0.01495 | | | |  | | 0.819 | | |  | 0.413 | | |  |
| College_Student: | | | | |  |  | | |  |  | | | |  | |  | | |  |  | | |  |
| Yes – No | | | | |  | 0.3091 | | |  | 0.26800 | | | |  | | 1.153 | | |  | 0.249 | | |  |
| Birth | | | | |  | -0.0107 | | |  | 0.00862 | | | |  | | -1.240 | | |  | 0.215 | | |  |
| Gender: | | | | |  |  | | |  |  | | | |  | |  | | |  |  | | |  |
| Men – Women | | | | |  | -0.8818 | | |  | 0.24234 | | | |  | | -3.639 | | |  | < .001 | | |  |
| ᵃ Represents reference level | | | | | | | | | | | | | | | | | | | | | | | |
|  | | | | | | | | | | | | | | | | | | | | | | | |

**Regression - Control Frequency**

| Model Fit Measures | | | | | | | | | | | | | | | | | | | | | | |  |
| --- | --- | --- | --- | --- | --- | --- | --- | --- | --- | --- | --- | --- | --- | --- | --- | --- | --- | --- | --- | --- | --- | --- | --- |
|  | | | | | | | | | **Overall Model Test** | | | | | | | | | | | | | |  |
| **Model** | | **R** | | | **R²** | | | | **F** | | | **df1** | | | | **df2** | | | **p** | | | |  |
| 1 | Control_Recoded | 0.0139 |  | | 1.94e-4 | |  | | 0.185 | |  | 1 | |  | | 953 |  | | 0.667 | |  | |  |
| 2 | Control_Recoded DRF_Recoded | 0.0373 |  | | 0.00139 | |  | | 0.662 | |  | 2 | |  | | 952 |  | | 0.516 | |  | |  |
| 3 | Control_Recoded DRF_Recoded  Age | 0.0547 |  | | 0.00299 | |  | | 0.950 | |  | 3 | |  | | 951 |  | | 0.416 | |  | |  |
| 4 | Control_Recoded DRF_Recoded  Age  College_Student: | 0.0713 |  | | 0.00508 | |  | | 1.214 | |  | 4 | |  | | 950 |  | | 0.303 | |  | |  |
| 5 | Control_Recoded DRF_Recoded  Age  College_Student  Gender | 0.1360 |  | | 0.01851 | |  | | 3.579 | |  | 5 | |  | | 949 |  | | 0.003 | |  | |  |
|  | | | | | | | | | | | | | | | | | | | | | | |  |
| Model Coefficients - PSQI_Total | | | | | | | | | | | | | | | | | | | | | | | |
|  | | | |  | |  | |  | |  | | |  | |  | | |  | |  | |  | |
| **Predictor** | | | | | | **Estimate** | | | | **SE** | | | | | **t** | | | | | **p** | | | |
| Intercept ᵃ | | | |  | | 30.17159 | |  | | 17.16509 | | |  | | 1.758 | | |  | | 0.079 | |  | |
| Control_Recoded | | | |  | | 0.00566 | |  | | 0.01848 | | |  | | 0.306 | | |  | | 0.760 | |  | |
| DRF_Recoded | | | |  | | 0.01212 | |  | | 0.01496 | | |  | | 0.810 | | |  | | 0.418 | |  | |
| College_Student: | | | |  | |  | |  | |  | | |  | |  | | |  | |  | |  | |
| Yes – No | | | |  | | 0.31333 | |  | | 0.26808 | | |  | | 1.169 | | |  | | 0.243 | |  | |
| Birth | | | |  | | -0.01089 | |  | | 0.00866 | | |  | | -1.257 | | |  | | 0.209 | |  | |
| Gender: | | | |  | |  | |  | |  | | |  | |  | | |  | |  | |  | |
| Men – Women | | | |  | | -0.87394 | |  | | 0.24257 | | |  | | -3.603 | | |  | | < .001 | |  | |
| ᵃ Represents reference level | | | | | | | | | | | | | | | | | | | | | | | |
|  | | | | | | | | | | | | | | | | | | | | | | | |

**Regression (Students Only)**

**Dream Recall Frequency**

| Model Fit Measures | | | | | | | | | | | | | | | | | | | |  |  |  |  |
| --- | --- | --- | --- | --- | --- | --- | --- | --- | --- | --- | --- | --- | --- | --- | --- | --- | --- | --- | --- | --- | --- | --- | --- |
|  | | | | | | **Overall Model Test** | | | | | | | | | | | | | |  |  |  |  |
| **Model** | | **R** | | **R²** | | **F** | | | | **df1** | | | **df2** | | | **p** | | | |  |  |  |  |
| 1 |  | 0.145 |  | 0.0209 |  | 1.92 | | |  | 3 |  | | 270 | |  | 0.127 | | |  |  |  |  |  |
|  | | | | | | | | | | | | | | | | | | | |  |  |  |  |
| Model Coefficients - PSQI_Total | | | | | | | | | | | | | | | | | | | | | | | |
|  | | | | | | |  |  | | | |  | |  | | |  |  | | |  |  |  |
| **Predictor** | | | | | | | | **Estimate** | | | | | | **SE** | | | | **t** | | | | **p** | |
| Intercept ᵃ | | | | | | |  | -128.0843 | | | |  | | 122.1002 | | |  | -1.049 | | |  | 0.295 |  |
| DRF_Recoded | | | | | | |  | 0.0481 | | | |  | | 0.0252 | | |  | 1.906 | | |  | 0.058 |  |
| Birth | | | | | | |  | 0.0681 | | | |  | | 0.0611 | | |  | 1.115 | | |  | 0.266 |  |
| Gender: | | | | | | |  |  | | | |  | |  | | |  |  | | |  |  |  |
| Une Femme – Un Homme | | | | | | |  | 0.2408 | | | |  | | 0.5178 | | |  | 0.465 | | |  | 0.642 |  |
| ᵃ Represents reference level | | | | | | | | | | | | | | | | | | | | | | | |
|  | | | | | | | | | | | | | | | | | | | | | | | |

**Regression (Students)**

**Lucid Dreaming Frequency**

| Model Fit Measures | | | | | | | | | | | | | | | | | |  |  |  |  |
| --- | --- | --- | --- | --- | --- | --- | --- | --- | --- | --- | --- | --- | --- | --- | --- | --- | --- | --- | --- | --- | --- |
|  | | | | | | **Overall Model Test** | | | | | | | | | | | |  |  |  |  |
| **Model** | | **R** | | **R²** | | **F** | | **df1** | | | | **df2** | | **p** | | | |  |  |  |  |
| 1 |  | 0.0918 |  | 0.00844 |  | 0.766 |  | 3 | |  | | 270 |  | 0.514 | | |  |  |  |  |  |
|  | | | | | | | | | | | | | | | | | |  |  |  |  |
| Model Coefficients - PSQI_Total | | | | | | | | | | | | | | | | | | | | | |
|  | | | | |  |  | | |  | |  | | | |  |  | | |  |  |  |
| **Predictor** | | | | | | **Estimate** | | | | | **SE** | | | | | **t** | | | | **p** | |
| Intercept ᵃ | | | | |  | -147.8155 | | |  | | 122.4913 | | | |  | -1.207 | | |  | 0.229 |  |
| Birth | | | | |  | 0.0781 | | |  | | 0.0613 | | | |  | 1.275 | | |  | 0.203 |  |
| Gender: | | | | |  |  | | |  | |  | | | |  |  | | |  |  |  |
| Une Femme – Un Homme | | | | |  | 0.3941 | | |  | | 0.5164 | | | |  | 0.763 | | |  | 0.446 |  |
| LDF_Recoded | | | | |  | -0.0360 | | |  | | 0.0814 | | | |  | -0.442 | | |  | 0.659 |  |
| ᵃ Represents reference level | | | | | | | | | | | | | | | | | | | | | |
|  | | | | | | | | | | | | | | | | | | | | | |

**Regression (Students)**

**Awareness frequency**

| Model Fit Measures | | | | | | | | | | | | | | | | | |  |  |  |  |
| --- | --- | --- | --- | --- | --- | --- | --- | --- | --- | --- | --- | --- | --- | --- | --- | --- | --- | --- | --- | --- | --- |
|  | | | | | | **Overall Model Test** | | | | | | | | | | | |  |  |  |  |
| **Model** | | **R** | | **R²** | | **F** | | **df1** | | | | **df2** | | **p** | | | |  |  |  |  |
| 1 |  | 0.0880 |  | 0.00774 |  | 0.702 |  | 3 | |  | | 270 |  | 0.552 | | |  |  |  |  |  |
|  | | | | | | | | | | | | | | | | | |  |  |  |  |
| Model Coefficients - PSQI_Total | | | | | | | | | | | | | | | | | | | | | |
|  | | | | |  |  | | |  | |  | | | |  |  | | |  |  |  |
| **Predictor** | | | | | | **Estimate** | | | | | **SE** | | | | | **t** | | | | **p** | |
| Intercept ᵃ | | | | |  | -145.14608 | | |  | | 124.6687 | | | |  | -1.1643 | | |  | 0.245 |  |
| Birth | | | | |  | 0.07681 | | |  | | 0.0624 | | | |  | 1.2317 | | |  | 0.219 |  |
| Gender: | | | | |  |  | | |  | |  | | | |  |  | | |  |  |  |
| Une Femme – Un Homme | | | | |  | 0.38390 | | |  | | 0.5163 | | | |  | 0.7436 | | |  | 0.458 |  |
| Awareness_Recoded | | | | |  | -0.00216 | | |  | | 0.0287 | | | |  | -0.0751 | | |  | 0.940 |  |
| ᵃ Represents reference level | | | | | | | | | | | | | | | | | | | | | |
|  | | | | | | | | | | | | | | | | | | | | | |

**Regression (Students)**

**Control frequency**

| Model Fit Measures | | | | | | | | | | | | | | | | | |  |  |  |  |
| --- | --- | --- | --- | --- | --- | --- | --- | --- | --- | --- | --- | --- | --- | --- | --- | --- | --- | --- | --- | --- | --- |
|  | | | | | | **Overall Model Test** | | | | | | | | | | | |  |  |  |  |
| **Model** | | **R** | | **R²** | | **F** | | **df1** | | | | **df2** | | **p** | | | |  |  |  |  |
| 1 |  | 0.0892 |  | 0.00796 |  | 0.722 |  | 3 | |  | | 270 |  | 0.539 | | |  |  |  |  |  |
|  | | | | | | | | | | | | | | | | | |  |  |  |  |
| Model Coefficients - PSQI_Total | | | | | | | | | | | | | | | | | | | | | |
|  | | | | |  |  | | |  | |  | | | |  |  | | |  |  |  |
| **Predictor** | | | | | | **Estimate** | | | | | **SE** | | | | | **t** | | | | **p** | |
| Intercept ᵃ | | | | |  | -146.36949 | | |  | | 122.5180 | | | |  | -1.195 | | |  | 0.233 |  |
| Birth | | | | |  | 0.07741 | | |  | | 0.0613 | | | |  | 1.263 | | |  | 0.208 |  |
| Gender: | | | | |  |  | | |  | |  | | | |  |  | | |  |  |  |
| Une Femme – Un Homme | | | | |  | 0.37132 | | |  | | 0.5176 | | | |  | 0.717 | | |  | 0.474 |  |
| Control_Recoded | | | | |  | 0.00840 | | |  | | 0.0325 | | | |  | 0.258 | | |  | 0.796 |  |
| ᵃ Represents reference level | | | | | | | | | | | | | | | | | | | | | |
|  | | | | | | | | | | | | | | | | | | | | | |

**Regression (General population)**

**Dream Recall Frequency**

| Model Fit Measures | | | | | | | | | | | | | | | | | | | |  |  |  |  |
| --- | --- | --- | --- | --- | --- | --- | --- | --- | --- | --- | --- | --- | --- | --- | --- | --- | --- | --- | --- | --- | --- | --- | --- |
|  | | | | | | **Overall Model Test** | | | | | | | | | | | | | |  |  |  |  |
| **Model** | | **R** | | **R²** | | **F** | | | | **df1** | | | **df2** | | | **p** | | | |  |  |  |  |
| 1 |  | 0.156 |  | 0.0243 |  | 5.60 | | |  | 3 |  | | 676 | |  | < .001 | | |  |  |  |  |  |
|  | | | | | | | | | | | | | | | | | | | |  |  |  |  |
| Model Coefficients - PSQI_Total | | | | | | | | | | | | | | | | | | | | | | | |
|  | | | | | | |  |  | | | |  | |  | | |  |  | | |  |  |  |
| **Predictor** | | | | | | | | **Estimate** | | | | | | **SE** | | | | **t** | | | | **p** | |
| Intercept ᵃ | | | | | | |  | 26.73771 | | | |  | | 17.73658 | | |  | 1.507 | | |  | 0.132 |  |
| DRF_Recoded | | | | | | |  | -0.00626 | | | |  | | 0.01855 | | |  | -0.338 | | |  | 0.736 |  |
| Birth | | | | | | |  | -0.00959 | | | |  | | 0.00895 | | |  | -1.072 | | |  | 0.284 |  |
| Gender: | | | | | | |  |  | | | |  | |  | | |  |  | | |  |  |  |
| Une Femme – Un Homme | | | | | | |  | 1.06530 | | | |  | | 0.27369 | | |  | 3.892 | | |  | < .001 |  |
| ᵃ Represents reference level | | | | | | | | | | | | | | | | | | | | | | | |
|  | | | | | | | | | | | | | | | | | | | | | | | |

**Regression (General population)**

**Lucid Dream Frequency**

| Model Fit Measures | | | | | | | | | | | | | |
| --- | --- | --- | --- | --- | --- | --- | --- | --- | --- | --- | --- | --- | --- |
|  | | | | | | **Overall Model Test** | | | | | | | |
| **Model** | | **R** | | **R²** | | **F** | | **df1** | | **df2** | | **p** | |
| 1 |  | 0.155 |  | 0.0241 |  | 5.56 |  | 3 |  | 676 |  | < .001 |  |
|  | | | | | | | | | | | | | |

| Model Coefficients - PSQI_Total | | | | | | | | | |
| --- | --- | --- | --- | --- | --- | --- | --- | --- | --- |
|  |  |  |  |  |  |  |  |  |  |
| **Predictor** | | **Estimate** | | **SE** | | **t** | | **p** | |
| Intercept ᵃ |  | 28.8037 |  | 16.77774 |  | 1.71678 |  | 0.086 |  |
| Birth |  | -0.0107 |  | 0.00845 |  | -1.26077 |  | 0.208 |  |
| Gender: |  |  |  |  |  |  |  |  |  |
| Une Femme – Un Homme |  | 1.0553 |  | 0.27216 |  | 3.87739 |  | < .001 |  |
| LDF_Recoded |  | 4.37e-4 |  | 0.04576 |  | 0.00955 |  | 0.992 |  |
| ᵃ Represents reference level | | | | | | | | | |
|  | | | | | | | | | |

**Regression (General population)**

**Awareness Frequency**

| Model Fit Measures | | | | | | | | | | | | | | | | | | | |  |  |  |  |
| --- | --- | --- | --- | --- | --- | --- | --- | --- | --- | --- | --- | --- | --- | --- | --- | --- | --- | --- | --- | --- | --- | --- | --- |
|  | | | | | | **Overall Model Test** | | | | | | | | | | | | | |  |  |  |  |
| **Model** | | **R** | | **R²** | | **F** | | | | **df1** | | | | **df2** | | **p** | | | |  |  |  |  |
| 1 |  | 0.158 |  | 0.0251 |  | 5.79 | | |  | 3 |  | | | 676 |  | < .001 | | |  |  |  |  |  |
|  | | | | | | | | | | | | | | | | | | | |  |  |  |  |
| Model Coefficients - PSQI_Total | | | | | | | | | | | | | | | | | | | | | | | |
|  | | | | | | |  |  | | | |  |  | | | |  |  | | |  |  |  |
| **Predictor** | | | | | | | | **Estimate** | | | | | **SE** | | | | | **t** | | | | **p** | |
| Intercept ᵃ | | | | | | |  | 28.7592 | | | |  | 16.66034 | | | |  | 1.726 | | |  | 0.085 |  |
| Birth | | | | | | |  | -0.0106 | | | |  | 0.00839 | | | |  | -1.264 | | |  | 0.207 |  |
| Gender: | | | | | | |  |  | | | |  |  | | | |  |  | | |  |  |  |
| Une Femme – Un Homme | | | | | | |  | 1.0588 | | | |  | 0.27198 | | | |  | 3.893 | | |  | < .001 |  |
| Awareness_Recoded | | | | | | |  | -0.0142 | | | |  | 0.01724 | | | |  | -0.823 | | |  | 0.411 |  |
| ᵃ Represents reference level | | | | | | | | | | | | | | | | | | | | | | | |
|  | | | | | | | | | | | | | | | | | | | | | | | |

**Regression (General population)**

**Control Frequency**

| Model Fit Measures | | | | | | | | | | | | | |
| --- | --- | --- | --- | --- | --- | --- | --- | --- | --- | --- | --- | --- | --- |
|  | | | | | | **Overall Model Test** | | | | | | | |
| **Model** | | **R** | | **R²** | | **F** | | **df1** | | **df2** | | **p** | |
| 1 |  | 0.156 |  | 0.0242 |  | 5.59 |  | 3 |  | 676 |  | < .001 |  |
|  | | | | | | | | | | | | | |

| Model Coefficients - PSQI_Total | | | | | | | | | |
| --- | --- | --- | --- | --- | --- | --- | --- | --- | --- |
|  |  |  |  |  |  |  |  |  |  |
| **Predictor** | | **Estimate** | | **SE** | | **t** | | **p** | |
| Intercept ᵃ |  | 29.32530 |  | 16.79021 |  | 1.747 |  | 0.081 |  |
| Birth |  | -0.01092 |  | 0.00846 |  | -1.292 |  | 0.197 |  |
| Gender: |  |  |  |  |  |  |  |  |  |
| Une Femme – Un Homme |  | 1.05306 |  | 0.27219 |  | 3.869 |  | < .001 |  |
| Control_Recoded |  | 0.00600 |  | 0.02248 |  | 0.267 |  | 0.790 |  |
| ᵃ Represents reference level | | | | | | | | | |
|  | | | | | | | | | |

**References**

**[1]** The jamovi project (2020). *jamovi*. (Version 1.2) [Computer Software]. Retrieved from <https://www.jamovi.org>.

**[2]** R Core Team (2019). *R: A Language and environment for statistical computing*. (Version 3.6) [Computer software]. Retrieved from <https://cran.r-project.org/>.
